# Interplay of demographics, geography and COVID-19 pandemic responses in the Puget Sound region: *The Vashon*, *Washington Medical Reserve Corps experience*

**DOI:** 10.1371/journal.pone.0274345

**Published:** 2023-08-16

**Authors:** James Bristow, Jamie Hamilton, John Weinshel, Robert Rovig, Rick Wallace, Clayton Olney, Vashon MRC COVID-19 Steering Committee, Karla J. Lindquist

**Affiliations:** 1 Vashon Medical Reserve Corps, Vashon, Washington, United States of America; 2 Island County Public Health Department, Coupeville, Washington, United States of America; 3 VashonBePrepared, Vashon, Washington, United States of America; 4 Atlas Genomics, Seattle, Washington, United States of America; 5 Madigan Army Medical Center, Joint Base Lewis McChord, Washington, United States of America; 6 Department of Epidemiology and Biostatistics, University of California San Francisco, San Francisco, California, United States of America; National Center for Global Health and Medicine, JAPAN

## Abstract

**Background:**

Rural U.S. communities are at risk from COVID-19 due to advanced age and limited access to acute care. Recognizing this, the Vashon Medical Reserve Corps (VMRC) in King County, Washington, implemented an all-volunteer, community-based COVID-19 response program. This program integrated public engagement, SARS-CoV-2 testing, contact tracing, vaccination, and material community support, and was associated with the lowest cumulative COVID-19 case rate in King County. This study aimed to investigate the contributions of demographics, geography and public health interventions to Vashon’s low COVID-19 rates.

**Methods:**

This observational cross-sectional study compares cumulative COVID-19 rates and success of public health interventions from February 2020 through November 2021 for Vashon Island with King County (including metropolitan Seattle) and Whidbey Island, located ~50 km north of Vashon. To evaluate the role of demography, we developed multiple linear regression models of COVID-19 rates using metrics of age, race/ethnicity, wealth and educational attainment across 77 King County zip codes. To investigate the role of remote geography we expanded the regression models to include North, Central and South Whidbey, similarly remote island communities with varying demographic features. To evaluate the effectiveness of VMRC’s community-based public health measures, we directly compared Vashon’s success of vaccination and contact tracing with that of King County and South Whidbey, the Whidbey community most similar to Vashon.

**Results:**

Vashon’s cumulative COVID-19 case rate was 29% that of King County overall (22.2 vs 76.8 cases/K). A multiple linear regression model based on King County demographics found educational attainment to be a major correlate of COVID-19 rates, and Vashon’s cumulative case rate was just 38% of predicted (p < .05), so demographics alone do not explain Vashon’s low COVID-19 case rate. Inclusion of Whidbey communities in the model identified a major effect of remote geography (-49 cases/K, p < .001), such that observed COVID-19 rates for all remote communities fell within the model’s 95% prediction interval. VMRC’s vaccination effort was highly effective, reaching a vaccination rate of 1500 doses/K four months before South Whidbey and King County and maintaining a cumulative vaccination rate 200 doses/K higher throughout the latter half of 2021 (p < .001). Including vaccination rates in the model reduced the effect of remote geography to -41 cases/K (p < .001). VMRC case investigation was also highly effective, interviewing 96% of referred cases in an average of 1.7 days compared with 69% in 3.7 days for Washington Department of Health investigating South Whidbey cases and 80% in 3.4 days for Public Health–Seattle & King County (both p<0.001). VMRC’s public health interventions were associated with a 30% lower case rate (p<0.001) and 55% lower hospitalization rate (p = 0.056) than South Whidbey.

**Conclusions:**

While the overall magnitude of the pre-Omicron COVID-19 pandemic in rural and urban U.S. communities was similar, we show that island communities in the Puget Sound region were substantially protected from COVID-19 by their geography. We further show that a volunteer community-based COVID-19 response program was highly effective in the Vashon community, augmenting the protective effect of geography. We suggest that Medical Reserve Corps should be an important element of future pandemic planning.

## Introduction

Vashon and Maury Islands (connected by a land bridge and together referred to as Vashon) form an island community of 10,953 located in unincorporated King County, near metropolitan Seattle, WA (S1 Fig). Located in Puget Sound, Vashon can be reached only by ferry and shares potential health risks from COVID-19 with other rural U.S. communities [1,2]. These risks include a median age of 54 with its attendant comorbidities, no local hospital, no weekend or acute care clinic, and a trip of up to 90 minutes to the nearest emergency room. Early in the pandemic, neither of Vashon’s two clinics offered COVID-19 testing. Vashon’s risk was compounded by a recent history of significant vaccine hesitancy [3].

Medical Reserve Corps (MRCs) are a network of ~800 volunteer medical organizations operating under the auspices of the Assistant Secretary for Preparedness and Response, in the U.S. Department of Health and Human Services, with the goal of strengthening public health, improving emergency response capabilities and building community resiliency [4]. In March of 2020, as the Seattle area became the nation’s first SARS-CoV-2 “hotspot” [5], the Vashon Medical Reserve Corps (VMRC) and VashonBePrepared, two community-based volunteer emergency preparedness organizations, established an integrated COVID-19 response program with the specific goal of reducing SARS-CoV-2 transmission in the Vashon community. This integrated program consisted of regular community engagement and education, free SARS-CoV-2 testing, rapid case investigation/contact tracing, an aggressive vaccination campaign and material support for affected community members. Over the first 22 months of the pandemic, Vashon had the lowest cumulative COVID-19 case rate in King County. In this study we investigate the contributions of demographics, geography and VMRC’s pandemic response program to Vashon’s low COVID-19 rates.

## Materials and methods

### Study design

To investigate the contributions of demographics, geography and the VMRC’s pandemic response program to COVID-19 cases, hospitalizations, and deaths, we carried out a retrospective cross-sectional comparison of cumulative COVID-19 rates from February 2020 through November 2021 across King County and Island County. Island County is comprised of geographically remote Whidbey Island and less remote Camano Island (S1 Fig). To delineate the contribution of demographics to COVID-19 rates, we created multiple linear regression models based on metrics of age, race/ethnicity, wealth, and education in 77 King County zip codes. To understand the contribution of remote geography on COVID-19 incidence, we expanded the regression models to include the demographically varied communities of North, Central and South Whidbey. Nearby Camano Island served as a non-remote regional control. The effectiveness of VMRC’s public health initiatives was evaluated through direct comparison of COVID-19 rates, vaccination rates and success of contact tracing on Vashon with King County and with South Whidbey, the Whidbey Island community with the greatest demographic and geographic similarity to Vashon.

### Public health responses

#### County responses

King and Island Counties were subject to the same statewide COVID-19 restrictions throughout the pandemic (S1 Table). Both counties carried out frequent public engagement through press releases, web postings and regular media coverage.

Public Health-Seattle & King County (PHSKC) emphasized on-demand testing throughout the study period and, in collaboration with the University of Washington, supported up to 10 free COVID-19 testing sites across the county beginning in April 2020. In addition, the City of Seattle partnered with Curative, Inc to support as many as nine additional test sites. In contrast, Island County Public Health (ICPH) primarily relied on healthcare providers to perform COVID-19 tests, who largely employed a testing strategy targeting symptomatic and exposed individuals. ICPH did perform 2500 on-demand tests over ~10 days in May 2020 to understand the prevalence of COVID-19, yielding no positive cases. As a result of their strategy, Island County tested at a rate just 15% of King County’s. However, Island and King Counties had similar ratios of hospitalizations to cases (5.6% for Island County vs 5.1% for King County). Because few COVID-related hospitalizations are likely to be missed, this suggests there was not a large undercount of Island County cases.

In both King and Island Counties, the great majority of vaccine doses were administered by pharmacies and health care providers. Both counties held vaccine clinics targeting at-risk communities.

PHSKC conducted its own case investigation/contact tracing throughout the study period [6]. A key component was linkage of contact tracing to available support services. ICPH performed contact tracing until November 2020, after which contact tracing was conducted by the Washington Department of Health (WADOH). Only Island County’s WADOH experience was available for analysis of contact tracing. Both PHSKC and WADOH relied upon laboratories to report cases through the WADOH Electronic Laboratory Reporting System, followed by generation of case reports and referral to contact tracers by the Washington Disease Reporting System [6].

#### Vashon MRC/VashonBePrepared response

The VMRC/VashonBePrepared pandemic response program was comprised of 3 primary efforts: community engagement, community health, and community support. The program was organized using a typical incident command structure led by an emergency operations center (S2 Fig). VMRC coordinated its activities with PHSKC and King County Emergency Management, but operated independently. Because Vashon does not meet the King County threshold for social vulnerability, King County did not provide or materially support testing or vaccination on Vashon.

The primary community engagement tool was “situation reports” created jointly by VashonBePrepared and VMRC. These were emailed to >3500 residents on weekdays in the first months of the pandemic, decreasing ultimately to twice weekly. Weekly summaries were also published in the local newspaper. These reports amplified and clarified frequently changing King County, Washington state and the federal Centers for Disease Control and Prevention guidance. VMRC operated a COVID-19 hotline 50 hours/week to provide individualized guidance and schedule SARS-CoV-2 tests. VMRC also advised more than 40 local businesses and schools about COVID-19 precautions and management of outbreaks.

During the study period, VMRC collected 5,705 COVID-19 samples at a drive-through site using supervised patient-collected nasal swab specimens [7]. This method was developed to minimize volunteer exposure to COVID-19 and the amount of personal protective equipment required. Testing targeted symptomatic patients and those with known exposures. SARS-CoV-2 PCR testing was carried out by a Clinical Laboratory Improvement Amendments (CLIA) approved, College of American Pathologists (CAP) accredited laboratory (Atlas Genomics, Seattle, WA). Results were usually returned within 24 hours of sample acquisition and always within 72 hours.

VMRC began contact tracing upon identification of its first case in June 2020 and ultimately investigated 87% of all Vashon cases during the study period. A team of four physicians delivered positive VMRC test results directly to patients by telephone within 24 hours of test completion, and immediately conducted interviews to identify close contacts, provide isolation and quarantine advice and refer for needed services through local Vashon or King County programs. Beginning in January 2021, PHSKC referred Vashon cases to VMRC for contact tracing. In July 2021, VMRC’s contact tracing protocol was streamlined to focus on household contacts, who comprised 75% of infected contacts up to that point.

After approval of COVID-19 vaccines in December 2020 [8], VMRC and VashonBePrepared, working with Vashon’s independent pharmacy, delivered 17,013 doses of SARS-CoV-2 vaccines- 75% of all doses delivered to Vashon residents. Vaccine administration occurred primarily at a drive through site (January-May 2021), outdoor walk-up clinics (June 2021) and indoor vaccine clinics held at a local church (October-November 2021). VMRC and VashonBePrepared also held clinics at local schools soon after school-age children became eligible (April-June 2021 and November 2021).

A COVID Relief Fund, run by VashonBePrepared and funded by individual charitable donations and Coronavirus Aid, Relief, and Economic Security (CARES) Act reimbursements, distributed $546,000 during the study period, primarily to local charities which in turn served community members. Additional details are in the caption of S2 Fig.

### Data sources and analysis

The study period extended from February 21, 2020 (the date of King County’s first COVID-19 case) through November 30, 2021, prior to the appearance of King County’s first Omicron cases. Daily COVID-19 cases, hospitalizations, deaths and test numbers for King County as a whole and by zip code were downloaded from the PHSKC COVID-19 dashboard [9]. Island County data were provided directly by ICPH. Daily vaccine administration data were provided by PHSKC and ICPH. Whidbey Island is home to a naval air station that fully vaccinated 8732 Navy employees, the majority of whom live off base. Calculation of Whidbey vaccination rates assumes these vaccine doses were administered over the course of 10 weeks beginning January 1, 2021.

Zip code level population data are the average of 2020 census zip code tabulation area estimates from the Washington State Office of Financial Management [10] and from Cubit (Austin, TX) based on the 2020 Decennial Census [11]. Age, race/ethnicity and educational attainment percentages, based on the 2020 American Community Survey [12], were also obtained from Cubit. The Asset Limited, Income Constrained, and Employed (ALICE) metric, a measure of the working poor, was obtained from United Way [13].

We developed a multiple linear regression model for cumulative COVID-19 case rates in the 77 King County zip codes (out of 120) with an estimated population greater than 1,000 that performed more than 500 tests/K population during the study period. To construct the model, we considered a variety of age, race/ethnicity, wealth, and educational metrics that might logically be related to COVID-19 case rates. The metric in each of these four categories with the highest R^2^ value in a simple linear regression of COVID-19 rates was then combined into a multiple linear regression model. The four metrics employed are the fraction of: 1) population <30 years of age; 2) population that is white or Asian (which have similar low COVID-19 rates in King County); 3) adult population with a bachelor’s degree; and 4) households below the ALICE threshold (S3 Fig). We used multiple linear regression with heteroscedasticity-consistent (HC3) robust standard errors [14] because the data showed modest deviation from normality and heteroscedasticity [15–17] (p<0.05). We calculated 95% prediction intervals from the multivariable model using robust standard errors of the point prediction for each observation. Multiple linear regression models of hospitalization and death rates were developed using the same variables and statistical methods.

For this study, we defined remote geography as an island community with <500 total vehicle trips/day/K population to or from the island. To assess population mobility on Vashon and Whidbey, we used Vashon and Whidbey Island ferry traffic data from the Washington State Department of Transportation (WSDOT) website [18] and calculated average daily vehicle and passenger traffic, normalized for population, by calendar quarter. We obtained estimates of quarterly bidirectional bridge traffic for Whidbey and Camano Islands directly from WSDOT. Because both Vashon and Whidbey have very little through traffic, passenger ferry traffic was converted to daily roundtrips by dividing daily passenger traffic by two. Passenger traffic over Whidbey’s bridge was estimated as the product of vehicle traffic and the quarterly average of passengers/vehicle on Whidbey ferries.

To assess the role of geography on COVID-19 rates, we expanded the linear regression models based on demographics to include a categorical variable for remote geography. Using the mobility data described above, we coded Vashon, North Whidbey, Central Whidbey and South Whidbey as remote. Camano Island was not coded as remote because its daily vehicle traffic exceeded 1000 vehicles/K population/day.

To directly evaluate the effectiveness of Vashon’s pandemic response program, we compared Vashon’s COVID-19 rates, the timeliness of vaccination, and success of case investigation and contact tracing with those of King County and South Whidbey, the Island County community with the greatest demographic similarity to Vashon. We compared the number of vaccine doses, normalized for population, administered over time using a Kolmogorov–Smirnov two-sample test to compare cumulative distributions [19]. We chose this metric because it reflects vaccination of the total at-risk population and is independent of changing eligibility requirements during the study period. We also calculated the time required to reach 1500 doses/K population, approximating 2 doses for 75% of population.

To examine the success and timeliness of contact tracing we compared the fraction of interviews that were successful, the test-to-interview interval, and number of close contacts identified by VMRC throughout the study period with available data from WADOH for South Whidbey Island (November 17, 2020 through November 30, 2021, including 90% of all South Whidbey cases) and with published data from PHSKC (July 2020 through June 2021, including 58% of all King County cases) [6]. Statistical significance was determined using Fisher’s Exact test with a Bonferroni correction for multiple comparisons.

The study protocol was reviewed and approved by the Human Subjects Protection Program at the University of California, San Francisco (#22–36518). Statistical comparisons were made with the Stata 17 software package (StataCorp LLC, College Station, TX). P-values <0.05 were considered statistically significant for all tests. Data from this study have been submitted to Dryad and are available without restriction [20]. The manuscript was prepared in accordance with STROBE guidelines [21].

## Results

### Demographic analysis of COVID-19 incidence in King County

**Fig 1:**
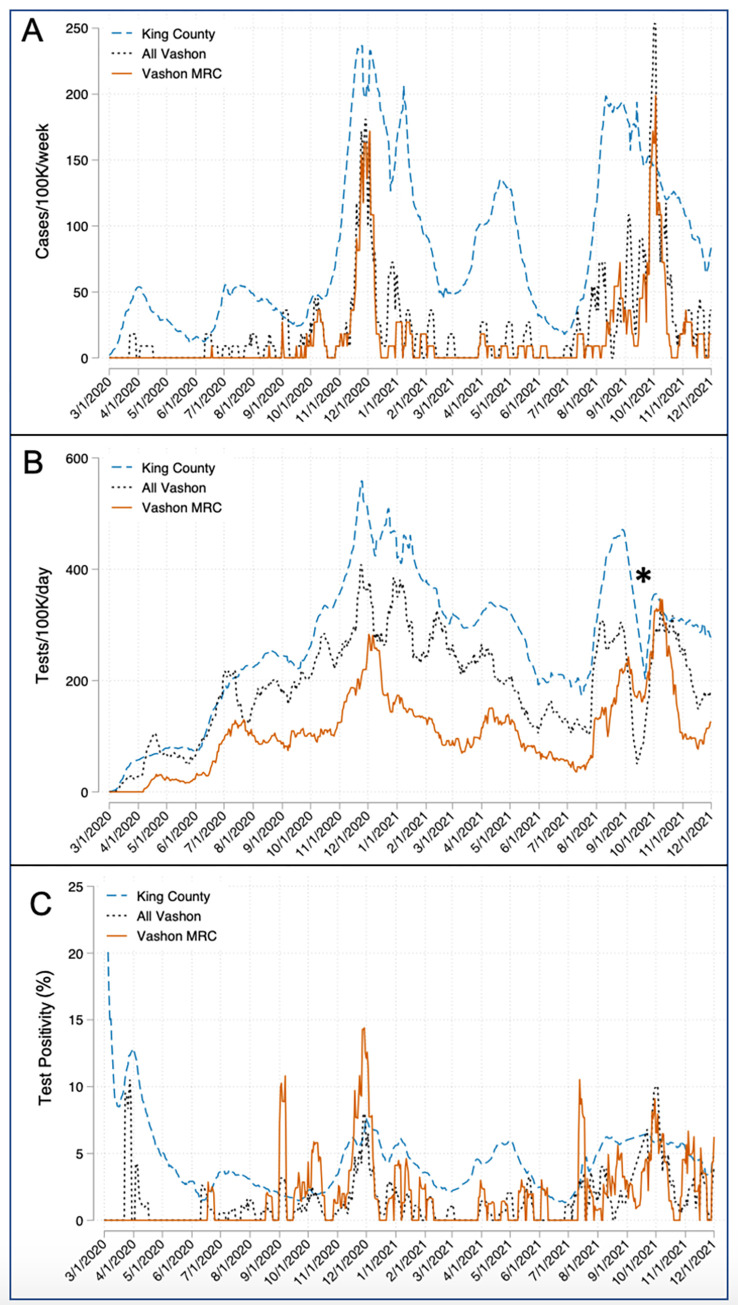
Chronology of SARS-CoV-2 cases, tests and test positivity rate for King County and Vashon. A) Case rates are presented as a rolling 7-day average normalized per 100K population. The Vashon case rates approached King County’s during the Beta surge in winter 2020 and briefy exceeded it during the Delta surge in winter 2021. B) Tests are presented as a 14-day rolling average to smooth the curves. Vashon MRC performed 41% of all Vashon tests but identified 65% of all Vashon cases. * Indicates a 2-week gap in WADOH test reporting that affected King County and All Vashon data, but not Vashon MRC. C) Test positivity rates are presented as 14-day rolling averages. The Vashon MRC positivity rate often exceeded King County’s during surges due to contact tracing activities.

As in much of rural America [22], the pandemic was delayed in reaching Vashon, but COVID-19 cases surged in November 2020, reaching 95% of the case rate in King County as a whole, and again in October 2021 when Vashon’s rate briefly exceeded the county rate (Fig 1). More than half of all Vashon’s cases observed during the study period occurred during these spikes. However, unlike many other rural communities and all of King County, Vashon’s case rate quickly declined after each surge, producing a cumulative case rate 71% lower than the King County rate during the study period (22.2 vs 76.8 cases/K). Vashon’s hospitalization and death rates were similarly lower than King County’s (0.73 vs 3.84/K and 0.37 vs 0.94/K respectively). VMRC conducted just 41% of all Vashon resident tests (Fig 1B), yet identified 65% of all Vashon cases. Test positivity rates largely remained below 5% (Fig 1C), suggesting Vashon’s testing rate was adequate to identify cases.

To understand the role of demographics in determining King County COVID-19 rates, we developed a multiple linear regression model of cumulative COVID-19 rates in 77 King County zip codes based on metrics of age, race/ethnicity, educational attainment, and wealth that showed the best correlations in a preliminary analysis (S3 Fig). Components of the model are the fractions of:

- Population age <30 years—the age group with the highest King County case rates
- Population that are white or Asian—groups with the lowest King County case rates
- Population age >25 years with a bachelor’s degree
- Households meeting ALICE criteria [11]—a measure of the working poor

**Fig 2:**
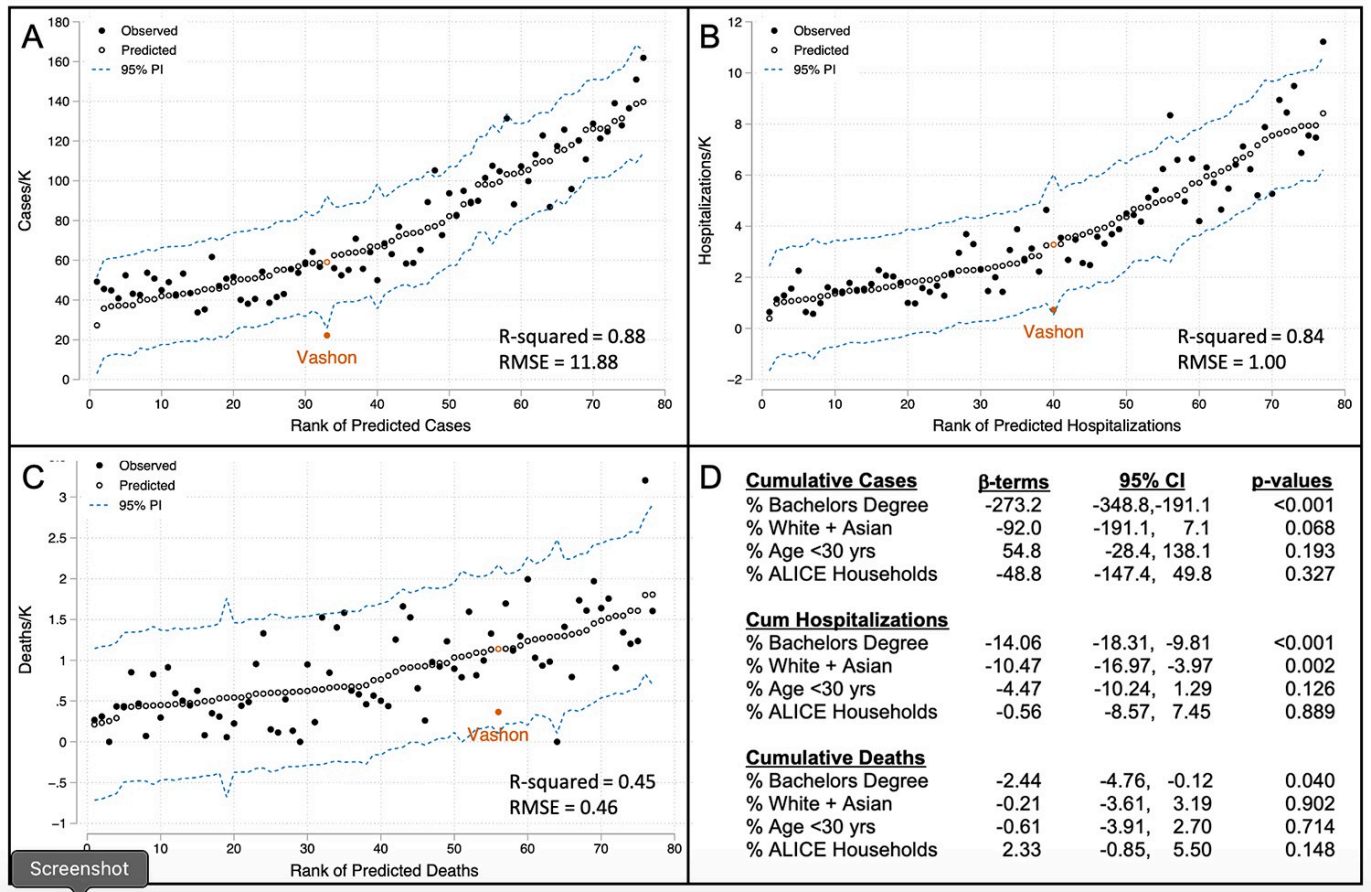
Models of cumulative incidence of COVID-19 cases, hospitalizations and deaths in King County from February, 2020 through November, 2021. Zip code-aggregated metrics of age, race/ethnicity, education and wealth were used to construct multiple linear regression models of cumulative incidence of COVID-19 cases (A), hospitalizations (B) and deaths (C). Open circles show predicted incidence in rank order and solid circles show observed incidence. Vashon data points are shown in gold. Beta-terms, confidence intervals and p-values for independent variables are shown in (D). 95% confidence intervals (CI) are calculated from robust HC3 standard errors. PI: Prediction interval; RMSE: Root mean squared error.

Predicted and observed cumulative COVID-19 rates for each zip code are shown in Fig 2, ranked by predicted value. Cumulative COVID-19 case rates varied 8-fold across King County and the regression model explains 88% of this variation. Vashon is a significant outlier, with an observed rate 38% of predicted (p < .05) and 32% lower than any other observed rate in King County. No King County zip code was predicted to have a cumulative case rate as low as Vashon’s observed rate.

We also modeled cumulative King County hospitalization rates and death rates (Fig 2B and 2C). Hospitalization rates closely paralleled the model of cases with a similar adjusted R^2^ value and beta-coefficients in the same relative order. Vashon’s predicted hospitalization rate was 4.5 times higher than observed (p = .07). Modeling of death rates showed more scatter with an R^2^ value of 0.45, due in part to the relatively small number of deaths in many zip codes. Interestingly, educational attainment is the dominant variable in all three models and the only variable that was significant in each (Fig 2D). Age and wealth metrics were surprisingly poor predictors of COVID-19 case, hospitalization, and death rates, but improved all three models by reducing standard errors, so they were retained.

### Contribution of remote geography to COVID-19 incidence in the Puget Sound region

**Fig 3:**
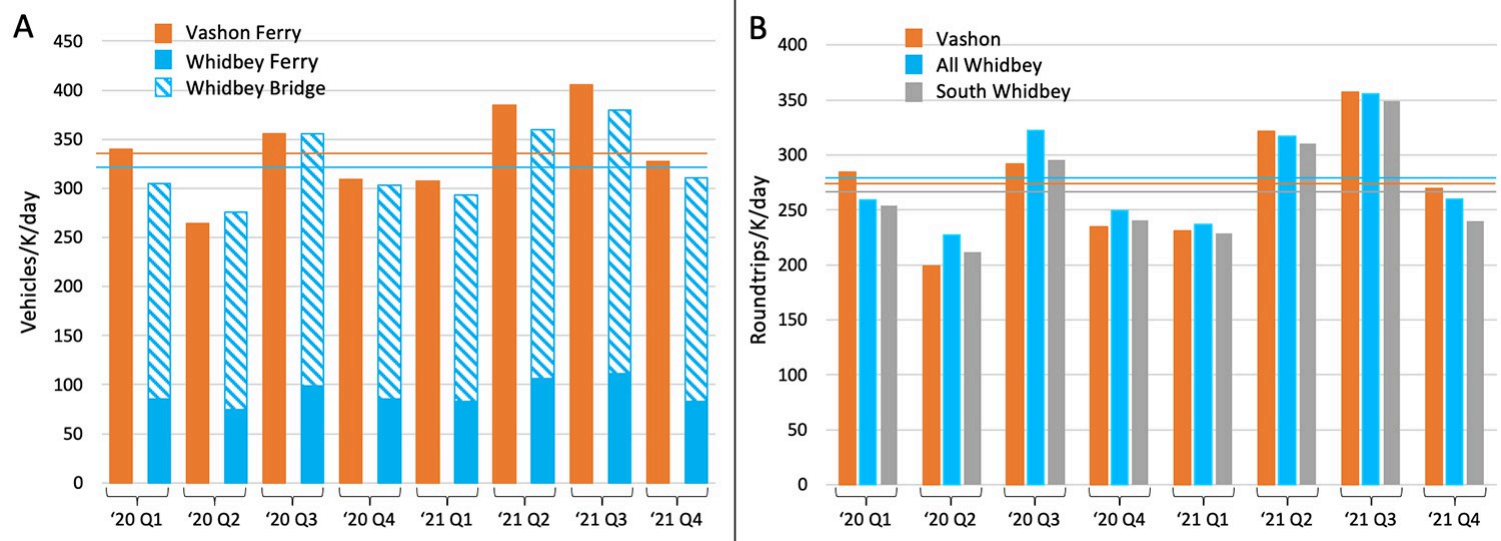
Mobility of Vashon and Whidbey Island populations. **A)** Daily total ferry traffic for Vashon, and road plus ferry traffic for Whidbey Island, are shown by quarter for 2020 and 2021, normalized for population. Averages for the study period are shown as horizontal lines in gold for Vashon and blue for Whidbey. While traffic varied significantly by quarter, Vashon and Whidbey’s rates were similar and highly correlated throughout the study period. B) Daily passenger roundtrips to/from Vashon and Whidbey, normalized for population, is shown by quarter. An estimate of South Whidbey passenger traffic, created as daily roundtrips on the Mulkiteo-Clinton ferry, normalized to the South Whidbey population, is also shown. Averages for the study period are shown in gold for Vashon, blue for Whidbey and gray for South Whidbey.

To understand the contribution of geography to Vashon’s low COVID rate, we expanded the analysis to include the communities of Whidbey Island, located ~50 km north of Vashon (S1 Fig). Like Vashon, Whidbey has very little through-traffic, allowing estimation of population mobility by analysis of vehicle traffic. As shown in Fig 3A, vehicle traffic, normalized for population, was very similar for Whidbey and Vashon throughout the study period. In contrast, Whidbey’s Island County neighbor, Camano Island, had average daily vehicle traffic of 1258 vehicles/K population/day during the study period, nearly 4 times that of Vashon or Whidbey. Daily passenger traffic to and from Vashon and Whidbey was likewise very similar throughout the study period (Fig 3B; averaging 273 vs 278 roundtrips/K population/day respectively).

Whidbey is demographically varied, so we divided Whidbey into 3 regions (North, Central and South; Table 1) for inclusion in the regression models and added a categorical variable that coded the three Whidbey communities and Vashon as “remote geography.” Camano Island, because of its greater vehicle traffic, was not coded as remote and serves as a control for regional variation independent of demographics and geography.

**Table 1:** Demographics of King County, Vashon Island, and Island County communities.

	King County	Vashon Island	South Whidbey	Central Whidbey	North Whidbey	Camano Island
**Population**	2,269,675	10,953	16,805	9,430	38,844	17,381
**Population//km** ^**2**^	406	109	98	87	266	166
**Median Income**	$99,158	$78,368	$83,471	$72,964	$61,373	$85,811
**ALICE Households**	21.2%	31.1%	20.1%	24.0%	22.8%	26.0%
**Median Age (years)**	37.0	54.2	58.2	59.8	33.6	54.8
**Age >60 years**	21.50%	38.4%	46.7%	50.1%	20.2%	41.6%
**Age <30 years**	37.5%	24.5%	21.2%	19.8%	44.2%	24.5%
**White, not Hispanic**	58.2%	84.1%	90.5%	89.7%	66.9%	89.7%
**Asian**	18.2%	2.0%	1.8%	0.8%	8.0%	2.4%
**Hispanic** ^**a**^	9.8%	5.7%	3.5%	5.2%	12.4%	3.5%
**Black**	6.4%	0.7%	1.0%	0.5%	3.8%	0.8%
**Amer. Indian/AK Native**	0.5%	0.2%	0.5%	1.3%	1.2%	0.4%
**Hawaiian/Pacific Islander**	0.7%	0.1%	0.1%	0.1%	0.7%	0.1%
**Multiracial/other**	6.1%	7.2%	2.6%	2.5%	7.0%	3.1%
**Bachelors Deg**	31.7%	29.4%	26.5%	19.6%	16.6%	22.2%

^a^In keeping with WADOH and American Community Survey reporting, Hispanic populations are coded as if it were a race. A non-white Hispanic person is therefore coded as Multiracial.

**Fig 4:**
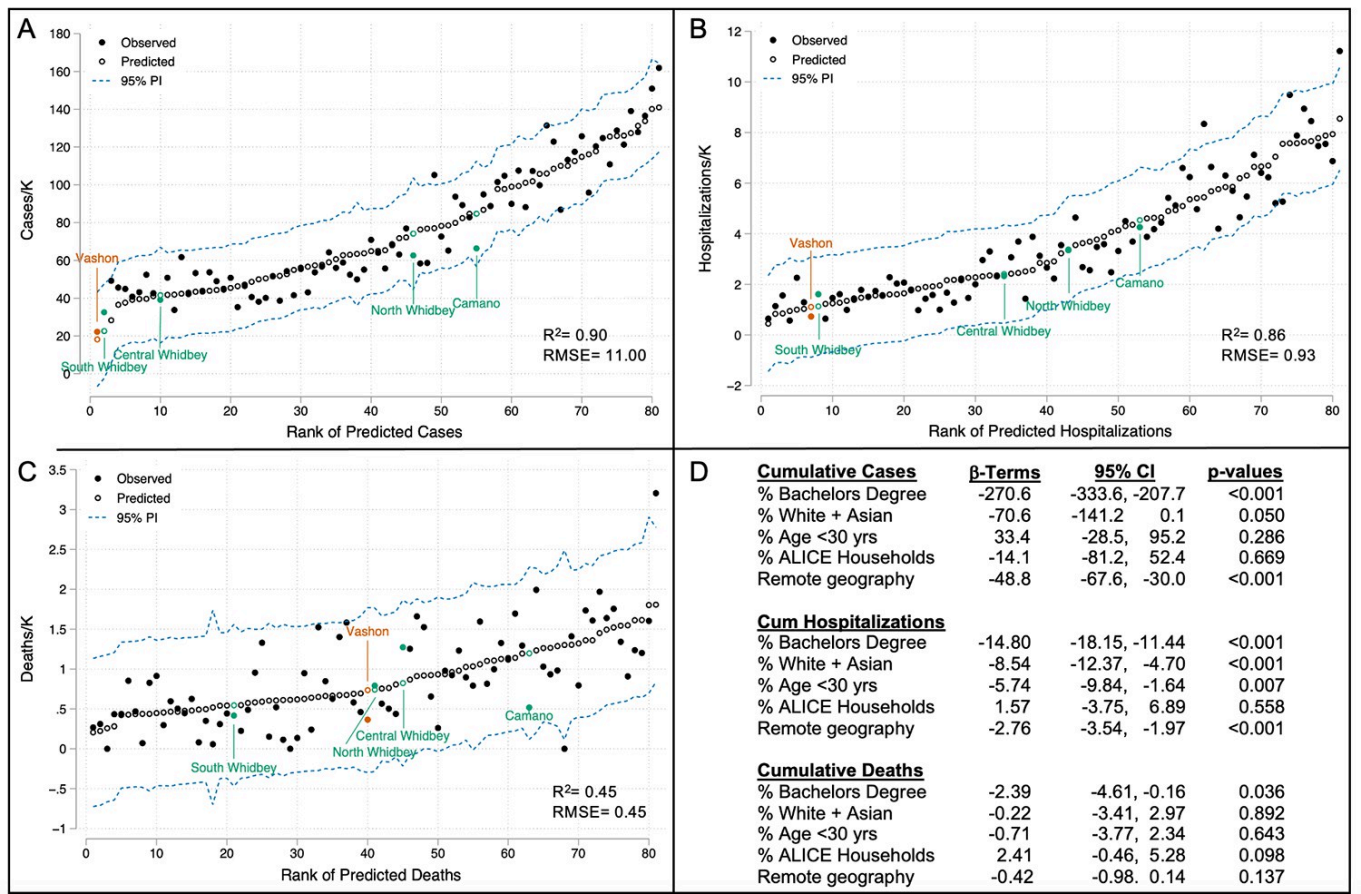
Models of cumulative incidence of COVID-19 cases, hospitalizations and deaths in the Puget Sound region from February, 2020 through November, 2021 including remote geography. Multiple linear regression models for cumulative incidence of cases (A), hospitalizations (B) and deaths (C) were expanded to include a categorical variable for remote geography. Open circles show predicted incidence in rank order and solid circles show observed incidence. Vashon data are shown in gold and Island County data are shown in green. Beta-coefficients, confidence intervals and p-values for independent variables are shown in panel D. PI: Prediction interval; RMSE: Root mean squared error.

Inclusion of remote geography improved the regression model of cumulative case rates by reducing the root mean squared error, placing all observed case rates above the 95% prediction interval and improving the p-value for the “White+Asian” variable (Fig 4). Despite significant mixing between Vashon and Whidbey populations with the mainland (Fig 3), the expanded regression model shows a large and highly significant negative contribution of remote geography to cumulative COVID-19 rates in the Puget Sound region: -49 cases/K (p<0.0001). Models for cumulative hospitalizations and deaths were similarly improved by inclusion of remote geography which reduced predicted hospitalizations by 2.8/K (p < .0001) and deaths by 0.42/K, although the beta-coefficient for the latter was not significant (p = 0.14).

### Impact of public health interventions on Vashon and Whidbey Island

While demographics and remote geography appear to explain much of Vashon’s reduced COVID-19 rates, the models’ predictions are less accurate at their extremes. In particular, observed case rates for the 9 zip codes with the lowest predicted rates (including Vashon and South Whidbey) all exceeded the predicted rate by an average of 26%. Because the regression models are insufficiently accurate to allow direct comparison of communities with similar demographics and geography, we evaluated the impact of public health measures through direct comparisons of cumulative COVID-19 rates, vaccination rates and success of contact tracing on Vashon and South Whidbey, Island County’s most demographically similar community to Vashon. South Whidbey’s population is older and less racially diverse than Vashon’s, but slightly less well-educated (Table 1), yielding similarly low predicted values in the regression models. Like Vashon, South Whidbey is accessed primarily by ferry and has a significant commuting population estimated to be similar to Vashon’s (Fig 3B). Finally, Vashon and Whidbey’s surrounding off-island counties have nearly identical population-weighted case rates (84 vs 87 cases/K respectively) so off-island exposure of residents is likely to be similar. For these reasons, South Whidbey appears to be an appropriate comparator to Vashon for evaluation of public health interventions.

**Fig 5:**
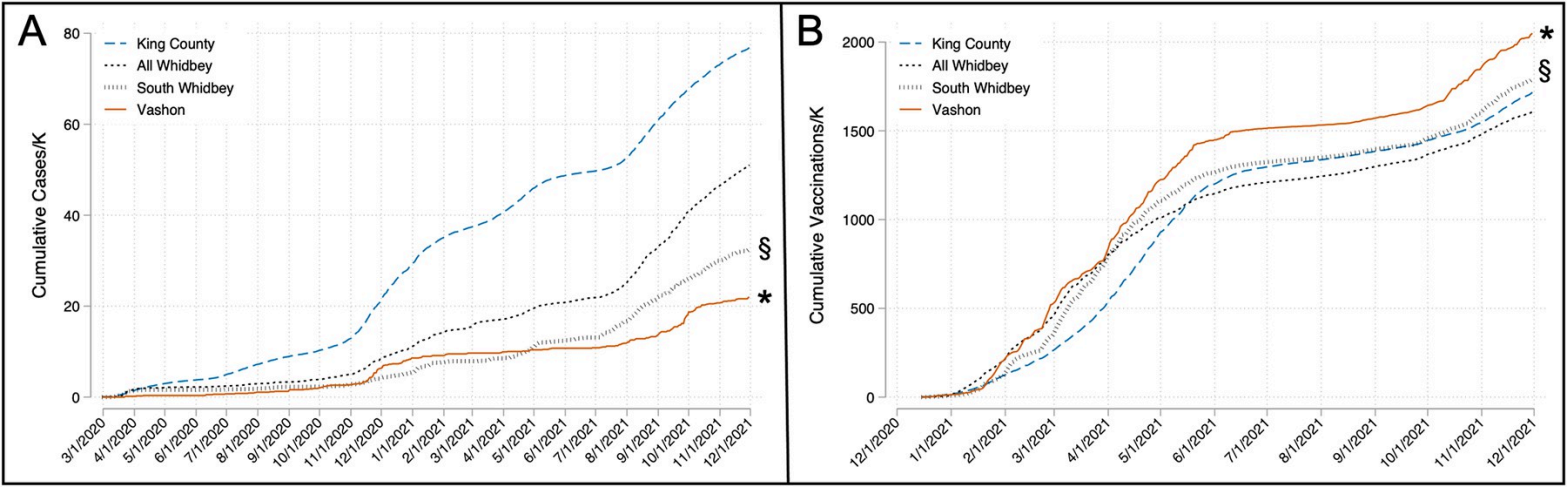
Cumulative cases and vaccinations for Vashon, South Whidbey, King County and all Whidbey. A) Cumulative cases normalized for population over time. B) Cumulative vaccination doses normalized for population. Vashon reached 1500 doses/K mnearly 4 months before King County or South Whidbey. §, Vashon vs King County and South Whidbey both p<0.001. *, South Whidbey vs all Whidbey p<0.001.

Cumulative COVID-19 case rates for King County, Vashon, all of Whidbey, and South Whidbey over the study period are shown in Fig 5A. As expected from their demographic profiles, Vashon and South Whidbey both had significantly reduced rates compared to King County and all of Whidbey (p < .001). Although Vashon and South Whidbey maintained similarly low cumulative case rates early in the pandemic, Vashon’s COVID-19 rate remained relatively flat after its November 2020 surge while South Whidbey’s rate continued to rise, ending the study period 45% higher than Vashon’s cumulative case rate (p<0.001). Vashon’s cumulative hospitalization rate was less than 50% of South Whidbey’s (0.73 vs 1.61/K) although this was of borderline significance (p = 0.056). Deaths were similar for both communities (0.36 vs 0.42/K).

Vaccination rates are compared in Fig 5B. Vashon and South Whidbey both were vaccinated at higher rates than surrounding communities (p<0.001), at least in part due to advanced median age that made a large fraction of these communities eligible for early vaccination. Despite its history of vaccine hesitancy [3], Vashon was vaccinated at a significantly accelerated pace compared to South Whidbey (p<0.001). VMRC and VashonBePrepared, working with the single island pharmacy, administered 75% of all vaccine doses given to Vashon residents. Vaccination in the region followed a predictable pattern with a rapid early rise followed by a long plateau beginning in summer of 2021. The Vashon plateau occurred at a level above 1500 doses/K population (a surrogate for 75% vaccination), a level only reached by South Whidbey and King County 118 days and 127 days after Vashon, respectively. From June through November 2021, the Vashon community averaged a vaccination rate 200 doses/K higher than South Whidbey.

To understand the impact of vaccination on cumulative COVID-19 rates, we repeated the multiple regression analysis including vaccination rate (doses/person) as a sixth variable (S4 Fig). Inclusion of vaccination rates was associated with a reduction in root mean squared error and reduced the impact of remote geography on cumulative case rates to -41 cases/K (CI: -58 to -23 cases/K, p<0.001). The effect on cumulative hospitalization and death rate models was negligible.

We also compared the effectiveness of case investigation/contact tracing for Vashon, South Whidbey, and King County as carried out by VMRC, WADOH and PHSKC, respectively (Table 2). For each group, no attempt was made to interview 12–15% of cases, but the reasons for this varied. For PHSKC, 5% were the result of reporting delay >14 days and 5% resulted from insufficient capacity during the winter surge of 2020. For South Whidbey, reporting delays accounted for all non-attempts. For Vashon, cases not tested by VMRC were not reported to VMRC prior to February 2021 (11%) and 2% were subsequently reported to VMRC by PHSKC >7 days after testing.

**Table 2:** Case investigation and contact tracing on Vashon, South Whidbey and in King County.

	VMRC/ Vashon	WADOH/ South Whidbey	PHSKC/ King County^a^
**Reporting period**	Jun ‘20-Nov ‘21	Nov ‘20-Nov ‘21	Jul’20-/Jun ‘21
**Cases in reporting period (% of all cases)** ^**b**^	237 (98%)	494 (90%)	61,269 (58%)
**Attempted interviews (% reported cases)**	206 (85%)	421 (85%)	53,917 (88%)
**Interviewed (% of attempted interviews)**	196 (95%)**	291 (69%)^c^	42,900 (80%)
**Could not reach (% of attempted)**	9 (4%)*	85 (20%)	NA
**Refused interview (% of attempted)**	1 (1%)	45 (11%)	NA
**Test-to-interview interval (IQR)**	1.7 days (1, 2)	3.7 days (2, 5)	3.4 days (NA)
**# Naming contacts (% of interviews)**	97 (49%)	124 (43%)	34,778 (81%)^§^
**# Contacts (avg # of contacts/interview)**	307 (1.57)	NA	77,385 (1.80)

^a^PHSKC data were previously reported [6].

^b^Reporting periods for VMRC, WADOH, and PHSKC represent all available data.

^c^Thirty-three successful South Whidbey interviews were conducted by submission of an online form. No information about close contacts was collected by these interviews, despite interviewees being asked about their contacts.

NA: Data were not made available. IQR: Interquartile range.

*Vashon vs South Whidbey p<0.001.

**Vashon vs South Whidbey & King County p<0.001.

^§^PHSKC vs VMRC and South Whidbey p<0.001.

Because VMRC contact tracers notified patients of a positive test by phone and conducted an interview immediately, they were successful in 95% of attempted interviews, compared with 80% of PHSKC attempted interviews and just 61% of WADOH/South Whidbey attempted interviews (both p<0.001 vs VMRC). Notably, 60% of failed VMRC interviews occurred in cases referred by PHSKC and <1% of Vashon interviews were refused, compared to 11% of WADOH/South Whidbey attempted interviews. VMRC also conducted interviews more quickly than PHSKC and WADOH, averaging 1.7 days, compared with 3.4 days for PHSKC and 3.7 days for WADOH.

**Fig 6:**
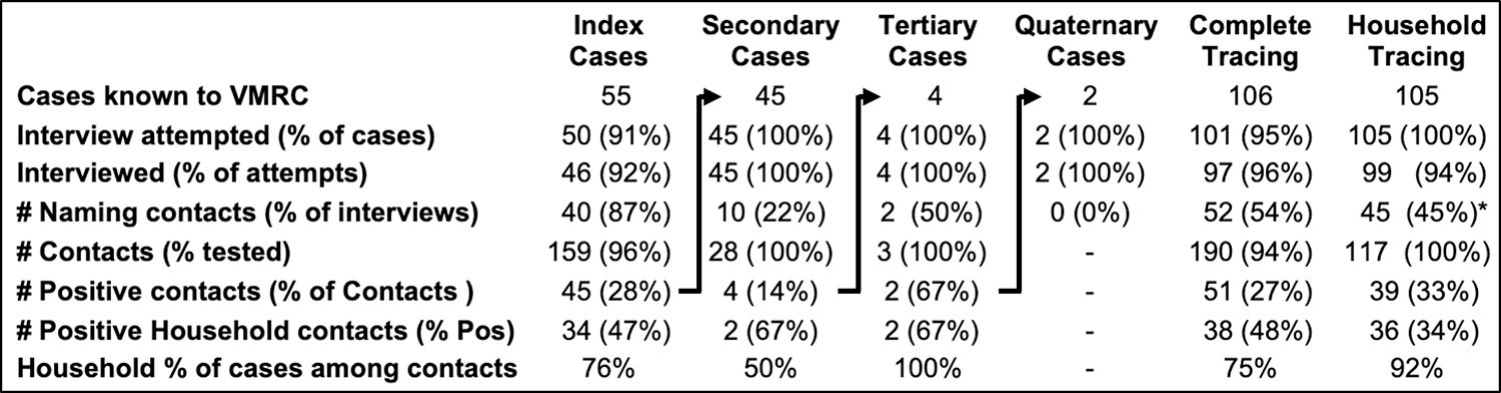
Structure of Vashon COVID-19 transmission. Data for all cases known to VMRC is shown. Arrows show transmission chain- contacts testing positive lead to the next generation of interviews tests and cases. The 1^st^ 55 index cases were asked to provide all contact information (Complete Tracing). In a 2^nd^ cohort, the focus shifted to identifying and testing household contacts (Household Tracing), although index patients were asked to notify contacts and have them call VMRC. *Sixty-eight percent of index cases in the Household Tracing cohort identified contacts.

While the fraction of interviewed cases naming contacts on Vashon and South Whidbey reflects the regional average [23], the difference between Vashon and PHSKC (49% vs 81%, p = .002) was of interest given Vashon’s success with public health measures in general and the overall response to contact tracing specifically. The reason for the discrepancy is apparent in the structure of Vashon community cases shown in Fig 6. VMRC index cases reported contacts with a frequency similar to PHSKC (85/106 or 80% of attempted interviews). Most subsequent cases were household contacts and/or contacts already in quarantine as a result of prior VMRC guidance, so that only 24% of subsequent cases had new contacts to name.

In VMRC’s initial cohort, household contacts were over four times more likely to test positive than non-household contacts (47% vs 11%). As a result, beginning in July 2021, VMRC focused its contact tracing effort exclusively on household contacts, resulting in very similar overall success rates.

## Discussion

Using multiple linear regression models, our study confirms the association of demographic factors with COVID-19 case, hospitalization, and death rates, as has been well-described by others [24–26]. In particular, our models show the significance of educational attainment as a predictor of case, hospitalization and death rates in the Puget Sound region. We speculate that educational attainment was a particularly strong regional indicator of those most able and willing to comply with public health measures, including working from home.

By extending the models to include rural island communities on Whidbey Island, we show that remote geography within a larger urbanized region is associated with an unexpectedly large reduction in the COVID-19 case rate. While lower population mobility is associated reduced COVID-19 transmission [27], the magnitude of this protective effect was surprising because residents of both Vashon and Whidbey Islands experience significant mixing with the mainland population. During the study period, 27–28% of the resident populations of Vashon and Whidbey made a daily roundtrip to or from the mainland (Fig 3B). This degree of mixing provided ample opportunity to introduce COVID-19 to both communities as demonstrated by the regular occurrence of cases on both islands throughout the study period (Figs 1 and 5A).

Empirical data exploring urban-to-rural epidemic gradients is quite limited beyond identifying the blessing of delayed pandemic arrival in isolated communities and the curse of subsequent explosive transmission in these communities that lack acute care capabilities [28]. Wells et al. [29] modeled urban-rural COVID-19 gradients in pre-vaccine Wales and concluded that the largest gradients are driven by high transmission rates in urban centers when test-and-trace strategies targeting both symptomatic and asymptomatic cases are in place. These factors allow better control of rural than urban COVID-19 transmission by contact tracing because of lower case burdens on the public health system. This scenario describes the Vashon and Whidbey environments at times of high urban transmission.

The importance of mobility for transmission of SARS-CoV-2 remains controversial. While early modeling [30] and empirical data [31] suggested mobility restrictions can limit transmission, in the context of the urban-rural gradient, mobility restrictions are predicted to increase urban cases and decrease rural ones without changing the overall size of the epidemic [29,32]. This model suggests that viral transmissibility and the success of other interventions to reduce transmission are more important factors than mobility or population density in setting up urban-rural pandemic gradients. We speculate that the protection afforded by remote geography that we observed is more likely related to these communities’ adherence to public health guidance than reduced mobility.

VMRC’s community-based public health interventions were highly effective and were associated with the lowest case rate and the 4^th^-lowest hospitalization rate in King or Island County, despite Vashon’s advanced median age. Direct comparison to South Whidbey, a geographically and demographically similar community, suggests that these efforts reduced Vashon’s case rate by 30% and hospitalization rate by 55%. Despite Vashon’s history of vaccine hesitancy [3], VMRC’s collaboration with a local pharmacy and coordinated public outreach enabled rapid vaccination of the island community, reaching 1500 doses/K (a proxy for 75% completion of the primary vaccine series) nearly 4 months earlier than South Whidbey and King County as a whole. VMRC case investigation and contact tracing also compares favorably with that conducted by PHSKC [6] and WADOH with respect to both successful interview rate and speed of investigation. VMRC was able to achieve this because 1) most test results were directly accessible to the contact tracing team rather than being reported to a state-wide system requiring subsequent referral to contact tracers, 2) positive results were delivered by contact tracers over the phone so interviews could begin immediately, and 3) the burden of cases was sufficiently small as to be manageable by the contact tracing team.

It is important to emphasize that PHSKC contact tracing was quite effective by national standards [23,33–36], especially considering the magnitude of their operation [6]. While less effective, WADOH contact tracing results represent the norm in the U.S. Nonetheless, comparison of the VMRC, PHSKC, and WADOH efforts suggests that decentralized, community-based programs may be an effective way of targeting contact tracing programs for at-risk communities.

Our study has limitations. First, the regression models presented were developed to fit local data with the specific goal of understanding the Puget Sound region’s COVID-19 rates. As such, they may not be generalizable to other regions. In particular, the effect of remote geography is based on a small number of observations and may not be reflective of other remote communities. Second, while we show that remote geography conferred protection in our region, “remoteness” may not be entirely responsible. A strong sense of community and trust in government institutions are essential components of successful pandemic responses both globally and locally [37,38], and were likely engendered through VMRC’s public engagement, material support, and amplification of public health messaging tuned to the Vashon community. This required close collaboration between VMRC and other volunteer units and community leaders (S2 Fig). Third, we cannot separate the contribution of the multiple elements of Vashon’s COVID-19 response program to its overall success. Integration of pandemic response elements is important [2] and VMRC managed that by providing Vashon residents with “one-stop shopping” for COVID-19 concerns. It is likely that communities that embrace testing, contact tracing, and vaccination also have greater acceptance of other non-pharmaceutical public health interventions.

Finally, it is not clear whether the VMRC program can be translated to other communities. VMRC clearly benefitted from pre-existing organizational expertise as well as a committed base of healthcare and other trained emergency workers with a broad range of professional experience. Many communities may lack this expertise. However, if provided with appropriate planning, protocols, and tools, we believe most communities could carry out a similar program with the support of a local clinic and/or their public health department- support that our community notably lacked. Elements of our program were successfully exported and implemented in several rural and tribal Pacific Northwest communities. At a larger scale, the Dell Medical School of the University of Texas at Austin, working with Austin Public Health, trained 281 student and community volunteers to conduct contact tracing [39]. Modeling based on their experience suggested that up to 78% of cases could be averted by accelerating the speed of contact tracing, as VMRC accomplished.

The activities we describe here are just one example of the under-reported effort from the nation’s ~800 Medical Reserve Corps units, most of which contributed in substantial ways to their communities’ pandemic response. We believe that MRCs can be particularly effective at extending the reach of local public health departments because they are community-based. Expanding the number of MRCs, strengthening connections with public health departments, and ensuring ongoing funding for training should be an essential element of U.S. pandemic planning.

## Supporting information

S1 Fig Geography of western Washington and the Puget Sound region. A) Map of western Washington showing selected counties of the Puget Sound region. The red boxed area is enlarged in panel B. B) The Puget Sound region. Major highways are shown in gold. The lower red-boxed area is enlarged in panel C (Vashon Island), and the upper red box is enlarged in panel D (Island County). Thick black lines in C and D indicate ferry routes. Note there is no bridge access to Vashon. In panel D, boundaries between North, Central and South Whidbey communities are shown by brown lines. Figures are redrawn and simplified from the U.S. Geological Survey and the Washington Geospatial Open Data Portals. Both are open-source resources. Maps are for illustrative purpose only.
(TIF)

S2 Fig Structure of the Vashon COVID-19 response program. VashonBePrepared is a 501(c)(3) organization that houses the Vashon Medical Reserve Corps (VMRC) and the Community Emergency Response Team for administrative and legal purposes. Following emergency activation by Vashon Island Fire & Rescue, the Vashon COVID-19 response assumed a typical incident command structure with operational control by the Emergency Operations Center. The Emergency Operations Center is staffed by volunteers from each of the 3 participating organizations. VashonBePrepared took primary responsibility for community engagement and material support; the VMRC had primary responsibility for the health-related activities and the Community Emergency Response Team had responsibility for logistics and support. During the study period membership of all three organizations grew, but none more than the MRC which began the pandemic with 9 members and grew to >100 over the course of the next year. VashonBePrepared raised more than $400,000 for its COVID-19 Relief Fund from hundreds of donors. Because some costs were reimbursed through CARES Act funding, the Relief Fund was ultimately able to distribute $546,000 in 4 areas: health, food security, housing security, and economic recovery. The testing and contact tracing effort that is the main thrust of this paper had a monthly cost of $1,200 as tests were largely paid by patient insurance. Uninsured patient tests were covered by the CARES Act or the COVID-19 Relief Fund. The Relief Fund supported food security by providing funding for the Vashon Maury Community Food Bank, the Vashon Senior Center and the Vashon Island School District nutrition program, resulting in the distribution of more than 25,000 meals and 4,300 bags of groceries. VashonBePrepared, working with the local Chamber of Commerce, also provided direct economic relief by helping 400 residents with applications for unemployment and other state or county benefits. The Relief Fund also provided emergency rent relief and other support to over 400 families needing assistance. Vashon’s Latino community received specific attention from these relief efforts.
(TIF)

S3 Fig Components of the multiple linear regression model for cumulative COVID-19 cases. Ordinary least squares linear regression of cumulative COVID-19 cases was carried out against several logical variables of age, race/ethnicity, educational attainment and wealth that might be associated with case rates in 77 King County zip codes. Those with the highest R^2^ values are shown: A) % of population with a Bachelor’s degree; B) % of population that is White or Asian; C) % of population of age <30 years; and D)% of households meeting ALICE criteria [12]. Total population, population density, and testing rate were also considered for inclusion in the model, but were not correlated with cumulative COVID-19 rates in King County during the study period.
(TIF)

S4 Fig Model of cumulative incidence of COVID-19 cases in the Puget Sound region from February, 2020 through November, 2021 including remote geography and vaccination. Beta-terms, confidence intervals and p-values for independent variables are shown in panel B. PI: Prediction interval; RMSE: Root mean squared error. Inclusion of vaccine doses administered/K population improves the R^2^ value and root mean squared error beyond that presented in Fig 4. This model continues to perform poorly at very low predicted values- 9 of the 10 lowest predicted case rates are exceeded by observed rates. Vashon and South Whidbey remain the lowest predicted case rates.
(TIF)

S1 Table Puget Sound pandemic timeline, 2020–2021. 1/21/20- 1^st^ U.S. case reported in Snohomish County, Washington 2/27/20- 1^st^ U.S. COVID-19 death reported in King County, Washington 2/29/20- Governor Inslee declared state of emergency. 3/13/20- Governor mandated statewide school closeures until April 24 & banned large events 3/16/20- PHSKC ordered no gatherings >50 or <50 if not meeting PHSKC standards 3/23/20- State issued 14-day “stay-at-home” order 4/2/20- Stay-at-home order extended until May 4 5/1/20- Governor Inslee announced announced phased “Safe Start” re-opening statewide 5/23/20- Island County approved for Phase 2 6/19/20- King County approved for Phase 2 6/21/20- Island County approved to move to Phase 3- 1^st^ in Puget Sound region to do so 6/26/20- Statewide mask mandate went into effect 11/16/20- State order: New statewide restrictions put in place until December 14, 2020 –no indoor gatherings, dining or fitness activities. 12/11/20- FDA authorizes Pfizer vaccine for people 16+ 12/12/20- Restrictions on gathering, dining & fitness extended until January 11, 2021 12/18/20- FDA authorized Moderna vaccine for people 18+ 12/27/20- FDA authorized Janssen (Johnson & Johnson) vaccine for people 18+ 1/12/21- Governor Inslee introduced new “Roadmap to Recovery” phased reopening plan 2/1/21- West region (including King, Snohomish and Island Counties) moved to Phase 2 5/10/21- FDA authorized Pfizer vaccine for adolescents 12–15 5/18/21- Statewide reopening to Phase 3 for all counties 5/30/21- Statewide reopening to Phase 4 9/22/21- FDA authorized Pfizer booster for people 65+ and people 18–64 in certain categories 10/20/21- FDA authorized Moderna and Johnson & Johnson booster for people 65+ and people 18–64 in certain categories 10/29/21- FDA authorized Pfizer vaccine for children 5–11 11/19/21- FDA authorized Moderna and Pfizer booster for adults 18+.
(DOCX)
