# Supplementary figures and images for: Interplay of demographics, geography and COVID-19 pandemic responses in the Puget Sound region: The Vashon, Washington Medical Reserve Corps experience
Source: PLoS One. 2023 Aug 16;18(8):e0274345. doi: 10.1371/journal.pone.0274345 (PMC10431654; doi:10.1371/journal.pone.0274345)

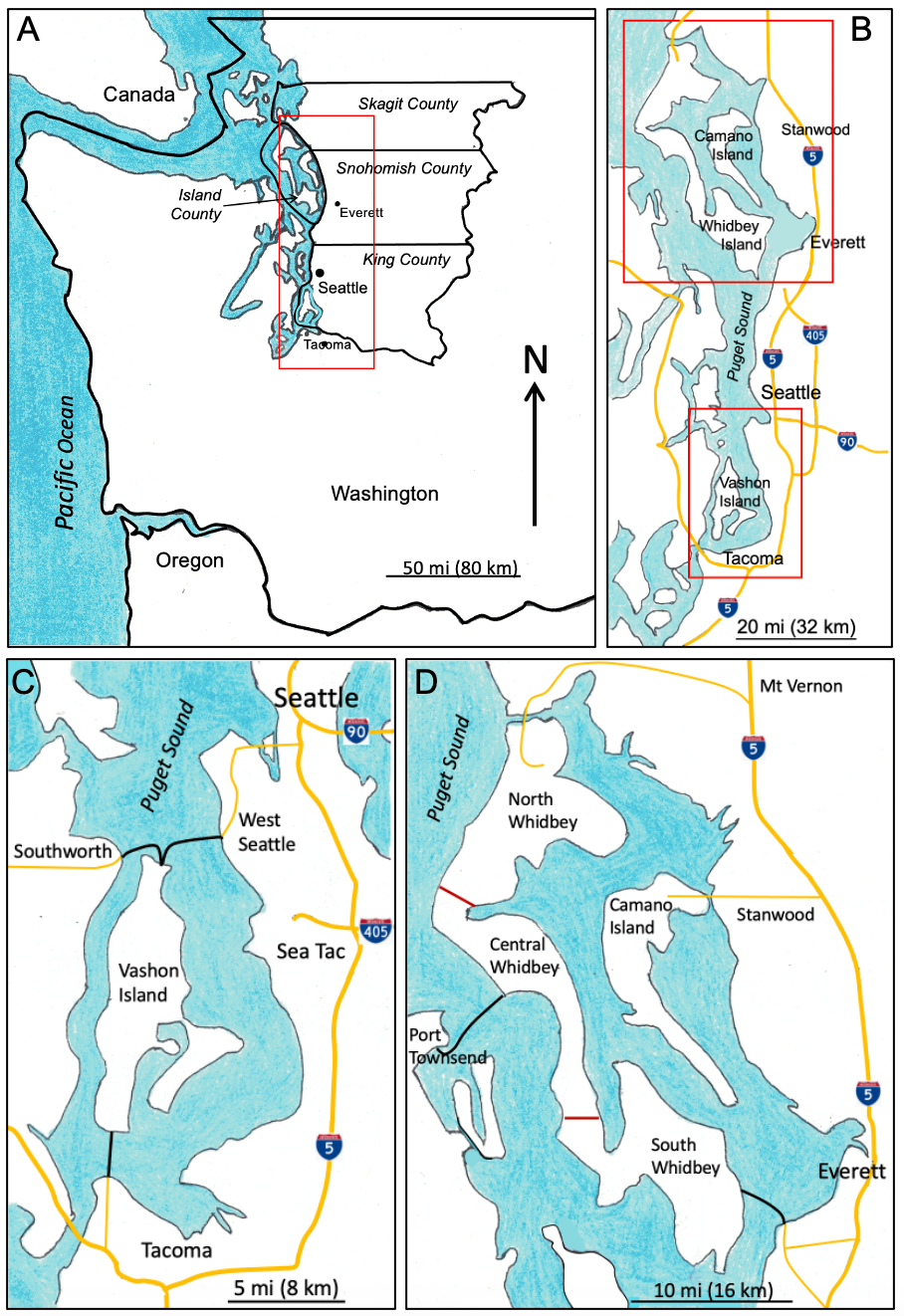

Supplement: S1 Fig — A) Map of western Washington showing selected counties of the Puget Sound region. The red boxed area is enlarged in panel B. B) The Puget Sound region. Major highways are shown in gold. The lower red-boxed area is enlarged in panel C (Vashon Island), and the upper red box is enlarged in panel D (Island County). Thick black lines in C and D indicate ferry routes. Note there is no bridge access to Vashon. In panel D, boundaries between North, Central and South Whidbey communities are shown by brown lines. Figures are redrawn and simplified from the U.S. Geological Survey and the Washington Geospatial Open Data Portals. Both are open-source resources. Maps are for illustrative purpose only. (TIF) [file pone.0274345.s001.tif]

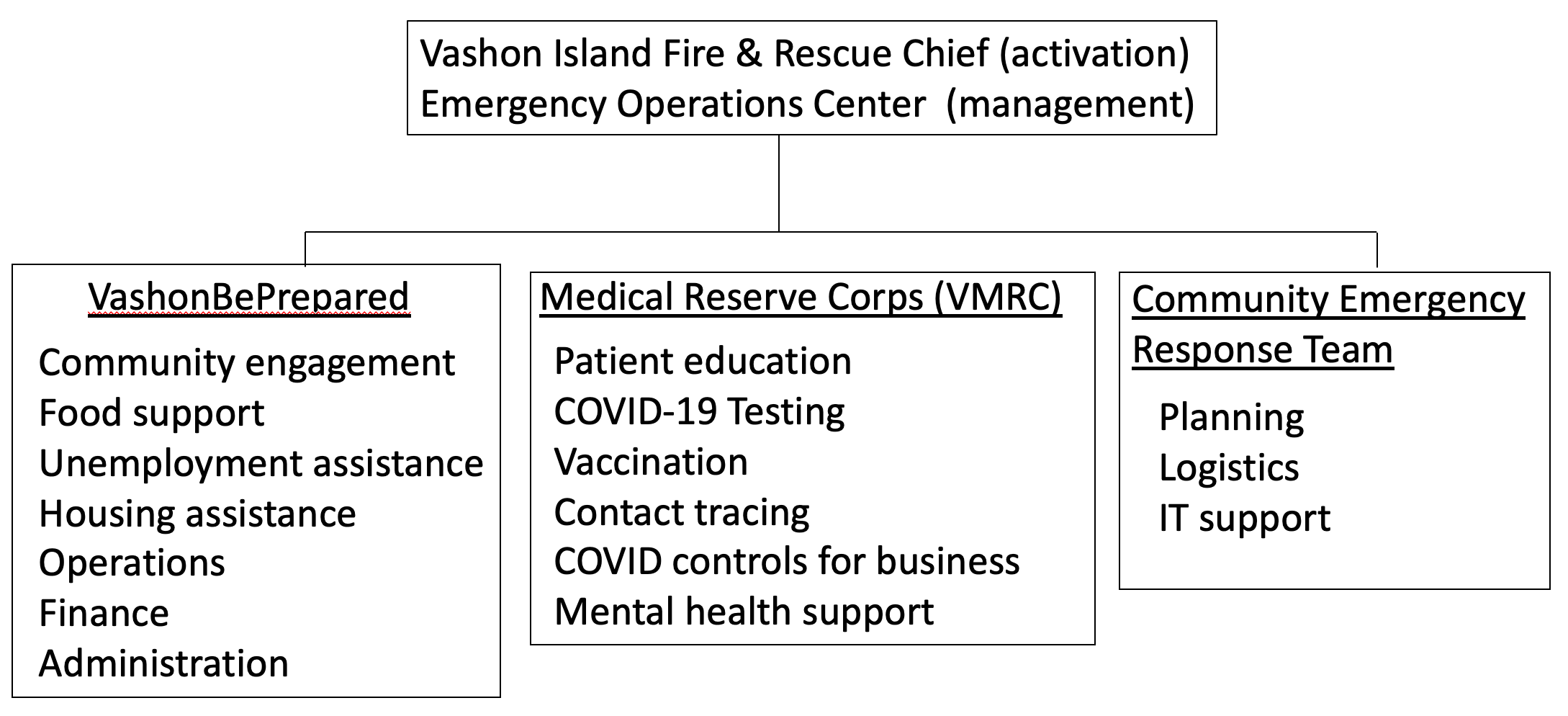

Supplement: S2 Fig — VashonBePrepared is a 501(c)(3) organization that houses the Vashon Medical Reserve Corps (VMRC) and the Community Emergency Response Team for administrative and legal purposes. Following emergency activation by Vashon Island Fire & Rescue, the Vashon COVID-19 response assumed a typical incident command structure with operational control by the Emergency Operations Center. The Emergency Operations Center is staffed by volunteers from each of the 3 participating organizations. VashonBePrepared took primary responsibility for community engagement and material support; the VMRC had primary responsibility for the health-related activities and the Community Emergency Response Team had responsibility for logistics and support. During the study period membership of all three organizations grew, but none more than the MRC which began the pandemic with 9 members and grew to >100 over the course of the next year. VashonBePrepared raised more than $400,000 for its COVID-19 Relief Fund from hundreds of donors. Because some costs were reimbursed through CARES Act funding, the Relief Fund was ultimately able to distribute $546,000 in 4 areas: health, food security, housing security, and economic recovery. The testing and contact tracing effort that is the main thrust of this paper had a monthly cost of $1,200 as tests were largely paid by patient insurance. Uninsured patient tests were covered by the CARES Act or the COVID-19 Relief Fund. The Relief Fund supported food security by providing funding for the Vashon Maury Community Food Bank, the Vashon Senior Center and the Vashon Island School District nutrition program, resulting in the distribution of more than 25,000 meals and 4,300 bags of groceries. VashonBePrepared, working with the local Chamber of Commerce, also provided direct economic relief by helping 400 residents with applications for unemployment and other state or county benefits. The Relief Fund also provided emergency rent relief and other support to over 400 fami [file pone.0274345.s002.tif]

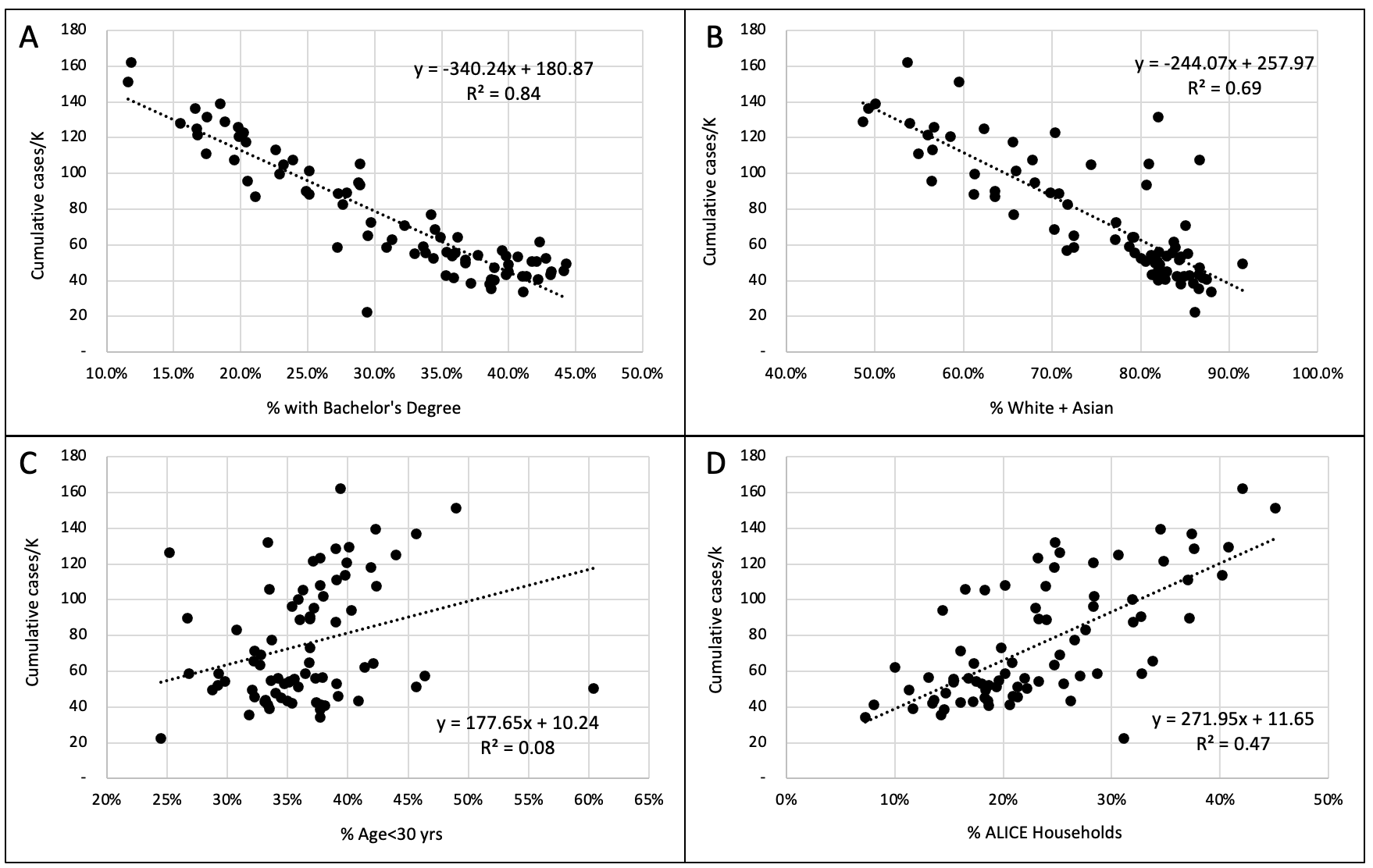

Supplement: S3 Fig — Ordinary least squares linear regression of cumulative COVID-19 cases was carried out against several logical variables of age, race/ethnicity, educational attainment and wealth that might be associated with case rates in 77 King County zip codes. Those with the highest R2 values are shown: A) % of population with a Bachelor’s degree; B) % of population that is White or Asian; C) % of population of age <30 years; and D)% of households meeting ALICE criteria [12]. Total population, population density, and testing rate were also considered for inclusion in the model, but were not correlated with cumulative COVID-19 rates in King County during the study period. (TIF) [file pone.0274345.s003.tif]

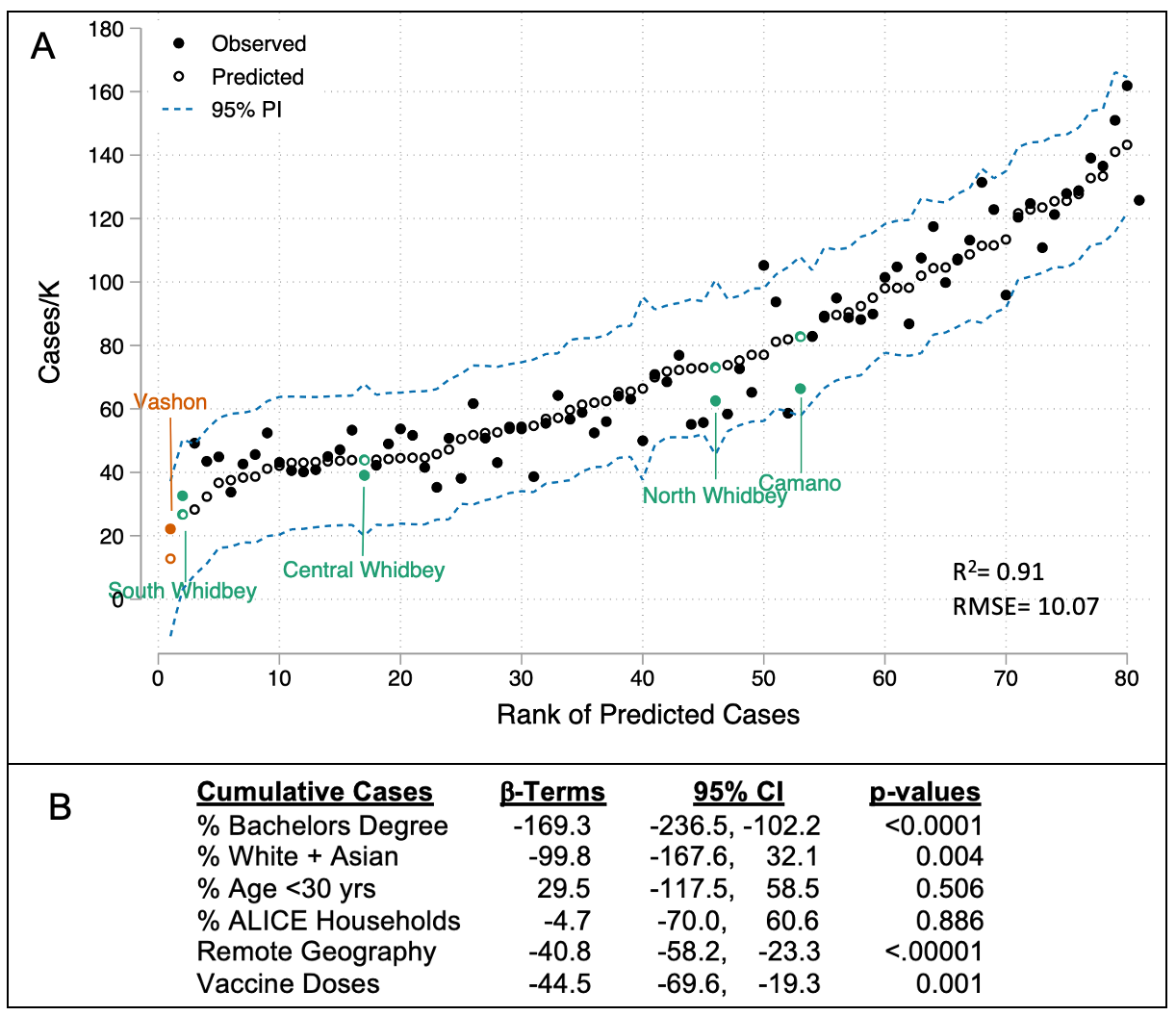

Supplement: S4 Fig — Beta-terms, confidence intervals and p-values for independent variables are shown in panel B. PI: Prediction interval; RMSE: Root mean squared error. Inclusion of vaccine doses administered/K population improves the R2 value and root mean squared error beyond that presented in Fig 4. This model continues to perform poorly at very low predicted values- 9 of the 10 lowest predicted case rates are exceeded by observed rates. Vashon and South Whidbey remain the lowest predicted case rates. (TIF) [file pone.0274345.s004.tif]
