# Supplementary material for: Interplay of demographics, geography and COVID-19 pandemic responses in the Puget Sound region: The Vashon, Washington Medical Reserve Corps experience
Source: PLoS One. 2023 Aug 16;18(8):e0274345. doi: 10.1371/journal.pone.0274345 (PMC10431654; doi:10.1371/journal.pone.0274345)
Supplement: S1 Table — 1/21/20- 1st U.S. case reported in Snohomish County, Washington 2/27/20- 1st U.S. COVID-19 death reported in King County, Washington 2/29/20- Governor Inslee declared state of emergency. 3/13/20- Governor mandated statewide school closeures until April 24 & banned large events 3/16/20- PHSKC ordered no gatherings >50 or <50 if not meeting PHSKC standards 3/23/20- State issued 14-day “stay-at-home” order 4/2/20- Stay-at-home order extended until May 4 5/1/20- Governor Inslee announced announced phased “Safe Start” re-opening statewide 5/23/20- Island County approved for Phase 2 6/19/20- King County approved for Phase 2 6/21/20- Island County approved to move to Phase 3- 1st in Puget Sound region to do so 6/26/20- Statewide mask mandate went into effect 11/16/20- State order: New statewide restrictions put in place until December 14, 2020 –no indoor gatherings, dining or fitness activities. 12/11/20- FDA authorizes Pfizer vaccine for people 16+ 12/12/20- Restrictions on gathering, dining & fitness extended until January 11, 2021 12/18/20- FDA authorized Moderna vaccine for people 18+ 12/27/20- FDA authorized Janssen (Johnson & Johnson) vaccine for people 18+ 1/12/21- Governor Inslee introduced new “Roadmap to Recovery” phased reopening plan 2/1/21- West region (including King, Snohomish and Island Counties) moved to Phase 2 5/10/21- FDA authorized Pfizer vaccine for adolescents 12–15 5/18/21- Statewide reopening to Phase 3 for all counties 5/30/21- Statewide reopening to Phase 4 9/22/21- FDA authorized Pfizer booster for people 65+ and people 18–64 in certain categories 10/20/21- FDA authorized Moderna and Johnson & Johnson booster for people 65+ and people 18–64 in certain categories 10/29/21- FDA authorized Pfizer vaccine for children 5–11 11/19/21- FDA authorized Moderna and Pfizer booster for adults 18+. (DOCX) [file pone.0274345.s005.docx]

**S1 Table. Puget Sound pandemic timeline, 2020-2021**

1/21/20- 1^st^ U.S. case reported in Snohomish County, Washington

2/27/20- 1^st^ U.S. COVID-19 death reported in King County, Washington

2/29/20- Governor Inslee declared state of emergency.

3/13/20- Governor mandated statewide school closeures until April 24 & banned large events

3/16/20- PHSKC ordered no gatherings >50 or <50 if not meeting PHSKC standards

3/23/20- State issued 14-day “stay-at-home” order

4/2/20- Stay-at-home order extended until May 4

5/1/20- Governor Inslee announced announced phased “Safe Start” re-opening statewide

5/23/20- Island County approved for Phase 2

6/19/20- King County approved for Phase 2

6/21/20- Island County approved to move to Phase 3- 1^st^ in Puget Sound region to do so

6/26/20- Statewide mask mandate went into effect

11/16/20- State order: New statewide restrictions put in place until December 14, 2020 – no indoor gatherings, dining or fitness activities.

12/11/20- FDA authorizes Pfizer vaccine for people 16+

12/12/20- Restrictions on gathering, dining & fitness extended until January 11, 2021

12/18/20- FDA authorized Moderna vaccine for people 18+

12/27/20- FDA authorized Janssen (Johnson & Johnson) vaccine for people 18+

1/12/21- Governor Inslee introduced new “Roadmap to Recovery” phased reopening plan

2/1/21- West region (including King, Snohomish and Island Counties) moved to Phase 2

5/10/21- FDA authorized Pfizer vaccine for adolescents 12–15

5/18/21- Statewide reopening to Phase 3 for all counties

5/30/21- Statewide reopening to Phase 4

9/22/21- FDA authorized Pfizer booster for people 65+ and people 18-64 in certain categories

10/20/21- FDA authorized Moderna and Johnson & Johnson booster for people 65+ and people 18-64 in certain categories

10/29/21- FDA authorized Pfizer vaccine for children 5-11

11/19/21- FDA authorized Moderna and Pfizer booster for adults 18+
